# Molecular epidemiology, risk factors, and outcomes of carbapenem-resistant *Klebsiella pneumoniae* infection in a tertiary hospital in eastern China: for a retrospective study conducted over 4 years

**DOI:** 10.3389/fmicb.2023.1223138

**Published:** 2023-07-27

**Authors:** Jun Cheng, Dongmei Zhao, Xuejiao Ma, Jiabin Li

**Affiliations:** Department of Infectious Disease, The First Affiliated Hospital of Anhui Medical University, Hefei, Anhui, China

**Keywords:** carbapenem-resistant *Klebsiella pneumoniae*, epidemiology, sequence type 11, risk factors, outcomes

## Abstract

**Objectives:**

Carbapenem-resistant *Klebsiella pneumoniae* (CRKP) have been extensively disseminated worldwide, resulting in increased mortality. We performed a retrospective analysis of the epidemiology and risk factors for the outcome of CRKP infection in a general teaching hospital in China.

**Methods:**

A molecular and clinical study was conducted for 98 CRKP in a tertiary hospital from January 2013 to December 2016. Carbapenemase gene detection, pulsed-field gel electrophoresis (PFGE), and multilocus sequence typing (MLST) were performed. Logistic regression was also used to identify the risk factors associated with 30-day mortality.

**Results:**

The production of KPC carbapenemase was the main resistant mechanism, and KPC carbapenemase increased annually with a significant difference. However, the molecular outcome revealed the dominance and diversity in CRKP with 24 sequence types (STs) and 59 PFGE types (PTs). The ST11 CRKP strains, which showed a significant increasing trend year by year, were documented as predominant in our study. Additionally, the predominant ST11 CRKP corresponding to PT10 and PT15 continued to exhibit their characteristic patterns. Importantly, the newly identified PT09 and PT16 strains, corresponding to the ST11 lineage, were only discovered in 2016. Meanwhile, factors affecting 30-day mortality and ST11 proportionality with CRKP infection were assessed, and ST11, appropriate empirical treatment, and hospital stays were found to be independently associated with 30-day mortality.

**Conclusion:**

The ST11 CRKP strains played a dominant role in the process; however, the homology of these strains was polymorphic, and the advantage clusters were subject to changes through evolution. Furthermore, in addition to appropriate empirical treatment and hospital stays, ST11 CRKP was independently associated with 30-day mortality. To the best of our knowledge, this association was reported for the first time.

## 1. Introduction

Carbapenem-resistant *Klebsiella pneumoniae* (CRKP) has emerged as a major nosocomial pathogen and is increasingly being reported worldwide (Tzouvelekis et al., 2012; Tängdén and Giske, 2015; Logan and Weinstein, 2017). It has been associated with a high mortality rate ranging from approximately 16% to 70% (Ben-David et al., 2012; Petrosillo et al., 2013). Carbapenem resistance in *K. pneumoniae* is reportedly caused by either epidemic clones or the horizontal dissemination of mobile elements. Additionally, the predominant production of *K. pneumoniae* carbapenemase (KPC) contributes to the most important mechanism of carbapenem resistance in *K. pneumoniae* (Munoz-Price et al., 2013). Of epidemiological significance, the international spread of KPC-producing *K. pneumoniae* is primarily associated with a single multilocus sequence type (ST) or its related variants. In Europe and America, KPC-producing ST258 *K. pneumoniae* is regarded as one of the most successful multidrug-resistant nosocomial pathogens (Tzouvelekis et al., 2012). However, the international high-risk clone of *K. pneumoniae*, ST11, is frequently reported as a successful pathogen in causing infections in Asia (Qi et al., 2010; Andrade et al., 2011; Munoz-Price et al., 2013). Wang et al. (2018) reported a significant difference in mortality associated with KPC-positive ST11 *K. pneumoniae*, which exhibited high resistance to meropenem.

Indeed, available data regarding the distribution of these resistance determinants among CRKP isolates collected over a long period are still limited in China, and information on the relationship between molecular characteristics and clinical outcomes is also sparing. In our study, we aimed to describe the epidemiology of CRKP, focusing on the molecular characteristics of circulating strains and evaluating the proportion of strains that were susceptible to clinical outcomes.

## 2. Methods

### 2.1. Study population

A retrospective study was conducted on specimens isolated from hospitalized patients infected with CRKP bacteria. The study was conducted from January 2013 to December 2016 in a hospital with 2,800 beds. Medical charts were reviewed, and demographic and clinical information was collected. This study was approved by the ethics committee of the First Affiliated Hospital of Anhui Medical University.

### 2.2. Bacterial isolates and genotypic investigation of resistance

Non-duplicate *K. pneumoniae* isolates, which developed within 48 h of hospital admission, were collected. These isolates were subsequently characterized as hospital-acquired infections with resistance to imipenem, meropenem, or ertapenem. Identification and antimicrobial drug susceptibility testing were performed using automated systems, specifically VITEK^®^2 (bioMérieux, France). Organisms were considered resistant to carbapenems based on the guidelines set by Clinical Laboratory Standards Institute (2016). Polymerase chain reaction (PCR) for the common *bla*_KPC_ gene was carried out to identify the carbapenemase genes of all CRKP.

### 2.3. Molecular typing

To observe and characterize the clonal relationships among the *K. pneumoniae* isolates, pulsed-field gel electrophoresis (PFGE) was conducted on XbaI-digested genomic DNA using the CHEF-DRIII system (Bio-Rad, United Kingdom). Strain-relatedness analysis was performed based on the Dice coefficient (SD) for genetic relatedness using the BioNumerics software version. The SD values reflect the degree of similarity between PFGE images of different strains; they range from 0 to 1, where 0 represents completely different and 1 represents completely identical. Different bands were determined to be of different types. According to the similarity coefficient between each pair of images, the unweighted pair group average method (UPGMA) was employed to construct a cluster tree for clustering, as described in reference (Han et al., 2013). Concomitantly, multilocus sequence typing (MLST) was the most common technique. It is based on genetic variations in seven housekeeping genes (*rpoB, gapA, mdh, pgi, phoE, infB, and tonB*) that together provide a relative genetic profile. Then, CRKP isolates were assigned ST numbers according to the allelic profiles available in the Institute Pasteur database. These ST data can be further defined using eBURST.

### 2.4. Clinical characterization and definitions

Clinical data were collected through electronic medical records, which extracted patient information such as demographic characteristics, chronic underlying diseases, invasive procedures, disease status, antimicrobial therapy, and clinical outcomes such as 30-day mortality. (1) Infection: all patients were evaluated according to the criteria set by the Center for Disease Control and Prevention (CDC) to assess whether the infection was due to CRKP (Horan et al., 2008). (2) Emergency state was defined as life-threatening organ dysfunction, which was evaluated by an acute change in the total Sequential Organ Failure Assessment (SOFA) score of ≥2 (Seymour et al., 2016). (3) Empirical and definitive therapy: Treatment administered before the susceptibility testing results were available was characterized as empirical, whereas any treatment administered after the results were available (at least one active drug for ≥48 h) was characterized as definitive therapy. The former was divided into appropriate and inappropriate treatment in our study. Appropriate antibiotic treatment was defined as treatment with at least one agent for ≥48 h after the isolation of a clinical culture specimen to which the isolate demonstrated susceptibility *in vitro* (Zarkotou et al., 2011). Definitive treatment regimens were classified as monotherapy (treatment with one *in vitro* active agent) and combination therapy (treatment with two or more *in vitro* active agents), with the latter including combinations involving tigecycline or other drugs. (4) Clinical outcome: Infection-related mortality was defined as the proportion of patients who died solely as a direct consequence of the CRKP infection, without any other plausible explanation according to the local investigator's opinion. Therefore, 30-day infection-related mortality referred to deaths of patients occurring within 30 days from the onset of the CRKP infection.

### 2.5. Statistical analysis

Statistical analysis was performed using SPSS version 21 software (IBM Corp., Armonk, NY). Univariate analysis was performed to identify factors related to mortality and to identify the ST11 of CRKP. The chi-square test was performed to assess categorical variables, and Student's *t*-test was performed to assess continuous variables. A logistic regression model was employed to identify factors independently associated with 30-day mortality. Sets of variables that had a *p*-value of ≤ 0.1 in the univariate analysis for mortality were entered into the model. *p*-values were interpreted together with a 95% confidence interval (CI) for the logistic regression model. All tests were two-tailed, and a *p*-value of < 0.05 was considered statistically significant.

## 3. Results

### 3.1. Isolates

A total of 98 patients with CRKP infection were identified during the study period. The median age was 62 years, with nearly three-quarters being male. Furthermore, 42 patients (42.9%) were admitted to the intensive care unit (ICU). The 98 non-duplicate CRKP isolates were obtained from sputum samples (72.4%), blood cultures (13.3%), and other samples (14.3%), and the main sites of infection were the lungs and the blood system. Based on antibiotic resistance testing for these isolates, imipenem MICs ranged from 2 to 16 mg/ml (two intermediate isolates), meropenem MICs ranged from 4 mg/ml to 16 mg/ml, and ertapenem MIC was 2 mg/ml (imipenem and/or meropenem MIC ≥ 4g/ml, ertapenem MIC ≥2 g/ml). Antimicrobial alternatives to carbapenems demonstrated through the automated systems included the resistance rates of 100% to ampicillin/sulbactam and over 93% to ceftazidime, aztreonam, ciprofloxacin, and piperacillin/tazobactam. Amikacin, gentamicin, and levofloxacin showed resistance rates of 80.6%, 82.7%, and 88.78%, respectively. All isolates except for the two intermediate ones were susceptible to tigecycline. Of these isolates, 60 isolates (61.2%) yielded a clinical specimen with the predominant *bla*_*KPC*_ -positive CRKP, and 55 isolates (56.1%) were classified into ST11. During the three periods from 2013 to 2016, we found that the CRKP infection had no statistical significance in the distribution of age, sex, ICU admission, and sample source. However, the CRKP isolates carrying *bla*_*KPC*_ showed an increasing trend year by year, which showed significant differences between the three periods (*p* < 0.001). Accordingly, a significantly higher prevalence of isolates containing ST11 CRKP (*p* < 0.001) was also observed in the recent years (Table 1).

**Table 1:** Carbapenem-resistant *Klebsiella pneumoniae* case characteristics.

	**Total**	**2013–2014**	**2015**	**2016**	***p*-value**
	**(*****n*** = **98, %)**	**(*****n*** = **24, %)**	**(*****n*** = **36, %)**	**(*****n*** = **38, %)**	
Age (years) (mean ± SD)	61.58 ± 19.07	61.46 ± 22.16	63.49 ± 17.09	58.90 ± 19.06	0.728
Gender (male)	72 (73.5)	18 (75)	27 (75)	27 (71.1)	0.911
ICU of admission	42 (42.9)	10 (41.7)	16 (44.4)	16 (42.1)	0.971
**Specimen source**					
Sputum	71 (72.4)	17 (70.8)	26 (72.2)	28 (73.7)	0.945
Blood	13 (13.3)	3 (12.5)	4 (11.1)	6 (15.8)	
Others	14 (14.3)	4 (16.7)	6 (16.7)	4 (10.5)	
KPC-2	60 (61.2)	4 (16.7)	23 (63.9)	33 (86.8)	< 0.001
ST11	55 (56.1)	4 (16.7)	22 (61.1)	29 (76.3)	< 0.001

SD, standard deviation; ICU, intensive care unit; KPC, Klebsiella pneumoniae carbapenemase; ST, sequence type.

### 3.2. Characterization of CRKP isolates through molecular analysis

In this study, 24 different STs were identified among the 98 *K. pneumoniae* isolates by MLST analysis. The most dominant sequence type was ST11 (56.1%, 55/98), followed by ST23 (11.2%, 11/98), ST15, and ST86 (3.1%, 3/98). Each of the 6 STs belonged to 2 isolates, while 14 STs obtained 1 strain. Among these, 10 STs of strains produced by KPC β-lactamase, of which the predominant ST11 harbored 49 KPC-producing *K. pneumoniae* isolates (89.1%, 49/55). Furthermore, ST23 contained 4 KPC-positive isolates (36.4%, 4/11), and the remaining STs possessed only one such isolate.

**Figure 1:**
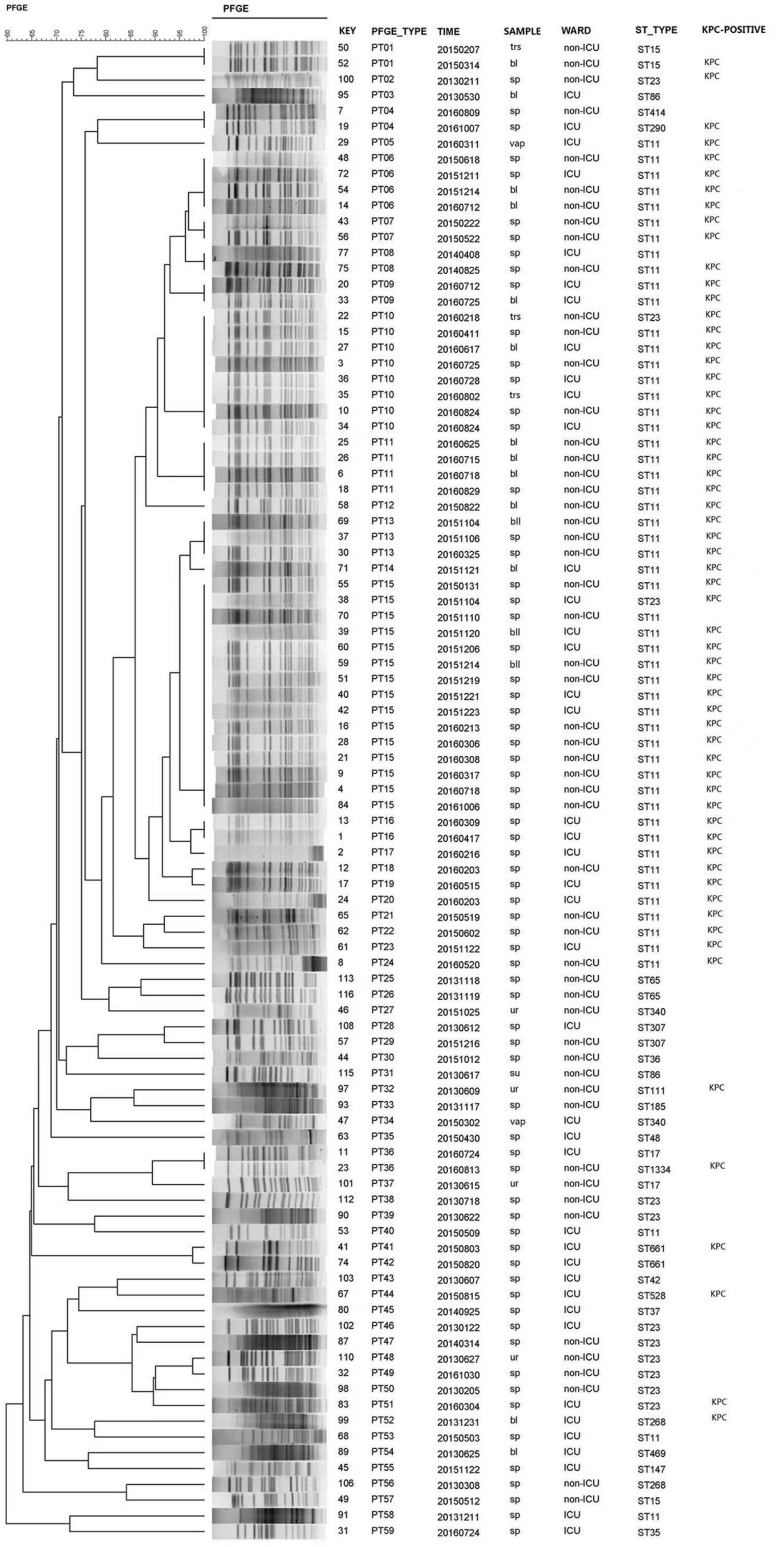
PFGE dendrogram generated using Bionumerics software showing the genetic relationship among 95 strains of carbapenem-resistant *Klebsiella pneumoniae*. The analysis of the bands generated was performed using the Dice similarity coefficient and the unweighted pair group method with arithmetic averages.

Of the 98 isolates, three isolates were ruled out due to chromosome nucleic acid degradation. Thus, 95 isolates were available for PFGE, and the analysis revealed 59 different clonal PFGE types (PTs, PT01-PT59). Of these isolates, the highest percentage clone was named PT15 (15.8%, 15/95), followed by PT10 (8.4%, 8/95), PT06 and PT11 (4.2%, 4/95), PT13 (3.2%, 3/95), and PT01, PT04, PT07, PT08, PT09, PT16, and PT36 (2.1%, 2/95). The remaining PTs possessed only one isolate. Interestingly, ST11 also occupied the predominant isolates according to the prepotent PFGE pattern. Additionally, 54 CRKP isolates (the actual was 55 strains for one nucleic acid degradation) of 23 PTs clones corresponded to the same type of ST11, and the dominant types of clones were PT15 (25.9%, 14/54), PT10 (13.0%, 7/54), PT06, and PT11 (7.4%, 4/54). According to clinical departments, 16 STs were observed in ICU admission. ST11 (57.1%, 24/42) and ST23 (7.1%, 3/42) were the most prevalent, harboring 25 (59.5%, 25/42) KPC-2-positive *K. pneumoniae* isolates. Furthermore, the dominant clones PT10 and PT15 corresponded to the predominant ST11 in the ICU. It is worth noting that only two strains of CRKP with the PT09 or PT16 clone type were recently discovered from ICU admission in 2016, which corresponded to ST11 (Figure 1).

### 3.3. Outcome and ST11

During the study period, 87 patients with CRKP infection were included in the analysis of clinical outcomes, and the remaining patients were excluded due to incomplete electronic information or due to their inability to follow up with the study. Among those with available outcome data, 32 patients (36.8%, 32/87) died within 30 days of the onset of the CRKP infection, while 55 patients (63.2%, 55/87) did not die within this time. To identify the potential risk factors for 30-day mortality, infected patients who died due to CRKP infection were compared with patients who survived (Table 2). The 30-day mortality was observed mainly in male patients (71.9%, 23/87) with a mean±standard deviation age of 60.19±21.31 years. A univariate analysis did not indicate any significant difference in sex, age, specimen source, underlying disease, emergency state, invasive operation, combination with other strains, or definitive therapy. However, the length of hospitalization was associated with 30-day mortality with a certain significance (*p* = 0.088) and so was admission to the ICU (*p* = 0.061). Notably, the 30-day mortality of patients that were administered appropriate empirical treatment was 9.4% (3/32), and the survival rate was 45.5% (25/55), which showed a significant difference (*p* < 0.001; Table 2).

**Table 2:** Univariate analysis of factors associated with 30-day mortality and ST11 for 87 patients with carbapenemase-resistant *Klebsiella pneumoniae* infection.

	**30-day-mortality (*n* = 32, %)**	**day-survivor (*n* = 55, %)**	***p*-value**	**ST11 (*n* = 53, %)**	**Non-ST11 (*n* = 34, %)**	***p*-value**
Gender, male	23 (71.9)	42 (76.4)	0.642	38 (71.7)	27 (79.4)	0.75
Age (years) (mean ± SD)	60.19 ± 21.31	63.73 ± 15.60	0.376	60.19 ± 19.22	65.91 ± 15.16	0.146
The length of hospitalization (mean ± SD)	32.5 ± 21.69	40.02 ± 29.35	0.088	37.87 ± 24.30	39.53 ± 31.24	0.782
ICU admission	17 (53.1)	18 (32.7)	0.061	24 (45.3)	11 (32.4)	0.23
**Specimen source**						
Sputum	22	41	0.421	40	23	0.261
Blood	6	5		8	3	
Others	4	9		5	8	
Underlying disease	24 (75)	42 (76.4)	0.886	39 (73.6)	27 (79.4)	0.535
Emergency state	23 (71.9)	35 (63.6)	0.432	40 (75.5)	18 (52.9)	0.03
Operative treatment	10 (31.3)	14 (25.5)	0.56	11 (20.8)	13 (38.2)	0.075
Invasive operation	5 (15.6)	15 (27.27)	0.213	14 (26.4)	6 (17.7)	0.343
Combination with other bacterium	23(71.9)	33 (60)	0.605	35 (66.0)	21 (61.8)	0.685
Appropriate empirical treatment	3 (9.4)	25 (45.45)	< 0.001	13 (25.4)	15 (44.1)	0.056
**Definitive therapy**						
Monotherapy	6	15	0.521	12	9	0.121
Combination with tigecycline	19	26		24	21	
Combination with carbapenem	7	14		17	4	

Due to the tendency of ST11 CRKP in the hospital mentioned above, we analyzed the possible risk factors depending on clinical data. We compared ST11 CRKP and non-ST11 CRKP, as presented in Table 2. Patients with ST11 CRKP infection had a significant emergency state compared to those with non-ST11 (*p* = 0.03). Additionally, both the operative and appropriate empirical treatment showed borderline significance with the ST11 CRKP infection (*p* = 0.075 or *p* = 0.056, respectively). Thus, no significant statistical difference in other related variables was found between the ST11 CRKP and non-ST11 strains.

A logistic regression analysis was conducted to investigate the association between 30-day mortality and various risk factors, including the length of hospitalization, ICU admission, appropriate empirical treatment, emergency state, and ST11 CRKP. The results revealed that the length of hospitalization [odds ratio (OR), 0.961; 95% confidence interval (CI) 0.935–0.988; *p* = 0.004], appropriate empirical treatment (OR, 11.301; 95% CI, 2.47–51.71; *p* = 0.002), and ST11 CRKP (OR, 0.193; 95% CI, 0.056–0.669; *p* = 0.01) were factors independently associated with 30-day mortality. Admission to the ICU and emergency state did not show significant statistical differences (Table 3).

**Table 3:** Factors associated with 30-day mortality in 87 patients with carbapenemase-resistant *Klebsiella pneumoniae* infection by logistic regression model.

**Variables**	**Odds ratio**	**95% confidence interval**		***p-*value**
		**Lower**	**Upper**	
The length of hospitalization	0.961	0.935	0.988	0.004
ICU admission	0.381	0.095	1.52	0.171
Emergency state	1.19	0.283	5.007	0.812
Appropriate empirical treatment	11.301	2.47	51.71	0.002
ST11	0.193	0.056	0.669	0.01

## 4. Discussion

Nosocomial infections due to CRKP are associated with substantial mortality, yet the clinical significance of isolating CRKP from the hospital is unknown. To our knowledge, this study is the first to describe the epidemiology and 30-day mortality of patients with CRKP infection through long-term retrospective observation. Our extensive research helped obtain several noteworthy findings. First, the trend of the KPC-positive CRKP strains belonging to the dominant ST11 lineage observed in this study provides further insight into the epidemiological characteristics. The outcome highlighted the diversity of CRKP strains, with 24 clones of ST and 59 clusters of PT. However, the strains of the predominant ST11 CRKP, corresponding to PT10 and PT15, remained prevalent. Importantly, the new advantage clusters of PT09 and PT16, which corresponded to ST11, were recently discovered from ICU admission in 2016. Second, the 30-day mortality of patients due to CRKP infection was associated with ST11 CRKP pathogens. In short, our findings provide new insights into the risk factors affecting clinical outcomes and further demonstrate the importance of epidemiology.

In our study, the investigation was conducted from 2013 to 2016 on CRKP isolates. Molecular epidemiological methods were employed to track and characterize CRKP isolates by PFGE and MLST, which were acceptable methods for differentiating the species—both of which identified CRKP ST11 as the predominant sequence type strain in our study. The epidemiology of CRKP is continually evolving, and the advantage strains of *bla*_*KPC*_-producing *K. pneumoniae* ST11, which is the most frequently detected KPC-producing *K. pneumoniae* clone in China, have been reported (Pitout et al., 2015; Sun et al., 2015; Liu J. X. et al., 2018). Interestingly, the dominance of ST11 among the isolates was also indicated by the prepotent PFGE pattern. Additionally, the dominant clones PT10 and PT15 corresponded to the advantage type strain of ST11 observed in the ICU, where they played an important role in the acquisition of carbapenem-resistant isolates (Katchanov et al., 2018). It is worth noting that the only two strains of CRKP with clone types PT09 or PT16, ranking only second to the dominant cluster PT, were recently discovered from ICU admission in 2016, and they corresponded to ST11. This study emphasized that, while ST11 CRKP played a dominant role in the process, the homology of these strains was polymorphic, and the advantage clusters were changed by evolution. Based on this, an efficient measure can be established to monitor the prevalence variation of these CRKP strains.

In this study, the overall 30-day mortality rate of patients infected with CRKP was 36.78% (32/87), which was similar to that reported by previous studies for CRKP infections (Chiu et al., 2017; Giannella et al., 2018). Most previous studies have concluded that a longer length of hospital stay, admission to the ICU, and exposure to antibiotics (penicillin/vancomycin and carbapenems) are associated with the development of CRKP infection (Liu et al., 2018; Zhu et al., 2020; Lou et al., 2022). Some studies have also shown that long-term hospitalization and empirical treatment are independent predictors of death (Tumbarello et al., 2012, 2015; Girometti et al., 2014). In our retrospective study, we observed that the length of hospitalization and appropriate empirical treatment were both independently associated with 30-day mortality. However, we could not provide further evidence for this. The primary objective of our study was to evaluate ST11 CRKP. Our discovery regarding the association between mortality and the pathogens of ST11 CRKP was unexpected, and to the best of our knowledge, we are the first to report this finding.

Next, we focused on the ST11 CRKP. The sequence types of globally prevalent carbapenem-resistant *Enterobacteriaceae*, including ST11 of *K. pneumoniae* clonal complex 258, were considered high-risk clones (Mathers et al., 2015). Thus, we presume that the analysis of the ST11 type and its association with clinical baseline factors are of significant concern. Although the underlying mechanisms behind the emergence of CRKP ST11 clones, as well as their associations with the outcomes, are yet to be elucidated, we reviewed the relevant literature and have attempted to provide some perspectives on the issue. Closely related to ST258, the factors of ST11 that contribute to its epidemiologic success remain unknown. However, several studies suggest that chromosomal or plasmid factors, beyond antibiotic resistance, may increase the strain's fitness and provide an advantage that underlies its prevalence (Chmelnitsky et al., 2012; Cottell et al., 2014). Additionally, a recent report showed that the KPC-producing *K. pneumoniae* ST11 clone was resistant to serum killing (Chiang et al., 2016), which may also be proof of the prevalence. In addition, a review explained that ST11 genome strains carried distinct capsular polysaccharide (cps) regions (Chen et al., 2014), and the cps locus was one of the primary determinants of antigenicity associated with *K. pneumoniae*. Capsule switching is a species-specific mechanism used by the microbe to escape the host immune response. Croucher et al. inferred that DNA exchange in and around the cps regions may be an important mechanism used by *K. pneumoniae* to rapidly diversify and evolve (Croucher and Klugman, 2014). Therefore, the successful prevalence of CRKP ST11 may be largely due to chromosomal recombination rather than antibiotic resistance. Brisse et al. (2009) found that strains owning some serotypes usually carried a relevant virulence factor content and have been associated with serious human infections, highlighting the potential threat represented by the emergence and diffusion of carbapenem-resistance clones with increased virulence potential (Zhang et al., 2015, 2016). Meanwhile, CRKP associated with plasmid-encoded carbapenemases can acquire multiclass antibiotic resistance to produce distinct clinical challenges and result in invasive infections with high mortality. Undoubtedly, careful monitoring of carbapenem susceptibilities and rapid identification of epidemiological lineages are quite necessary for implementing infection-control measures to prevent the endemicity of CRKP.

Our study has several limitations. Unlike previous observational studies, which were based on available data on patients with bacteremia or bacteriuria, our study did not unify patients with CRKP infection (Daikos et al., 2014; Qureshi et al., 2014; Tofas et al., 2016). Additionally, due to the limited number of strains acquired from the saved isolates in 2014, the study may be considered incomplete. Future research should investigate the virulent CRKP ST11 strains, which will allow us to expand our knowledge in this area.

In conclusion, ST11 CRKP played a dominant role in the process, but the homology of these strains was polymorphic, and the advantage clusters showed potential changes over time due to evolution. Additionally, apart from appropriate empirical treatment and hospital stays, ST11 CRKP was independently associated with 30-day mortality. Therefore, efforts are imminently required to improve our knowledge and understanding of the epidemiological status of CRKP to prevent its spread.

## Data availability statement

The original contributions presented in the study are included in the article/supplementary material, further inquiries can be directed to the corresponding author.

## Ethics statement

This study was approved by the Ethics Committee of The First Affiliated Hospital of Anhui Medical University (Quick- PJ 2017-11-19) and consent to participate.

## Author contributions

JL contributed substantially to the ideas and design. JC and DZ drafted the paper and responsible for the experiment. XM collected some clinical materials. All authors contributed to the article and approved the submitted version.
